# Tax-1 and Tax-2 similarities and differences: focus on post-translational modifications and NF-κB activation

**DOI:** 10.3389/fmicb.2013.00231

**Published:** 2013-08-15

**Authors:** Margret Shirinian, Youmna Kfoury, Zeina Dassouki, Hiba El-Hajj, Ali Bazarbachi

**Affiliations:** Department of Internal Medicine, Faculty of Medicine, American University of BeirutBeirut, Lebanon

**Keywords:** HTLV-1, HTLV-2, Tax-1, Tax-2, NF-κB

## Abstract

Although human T cell leukemia virus type 1 and 2 (HTLV-1 and HTLV-2) share similar genetic organization, they have major differences in their pathogenesis and disease manifestation. HTLV-1 is capable of transforming T lymphocytes in infected patients resulting in adult T cell leukemia/lymphoma whereas HTLV-2 is not clearly associated with lymphoproliferative diseases. Numerous studies have provided accumulating evidence on the involvement of the viral transactivators Tax-1 versus Tax-2 in T cell transformation. Tax-1 is a potent transcriptional activator of both viral and cellular genes. Tax-1 post-translational modifications and specifically ubiquitylation and SUMOylation have been implicated in nuclear factor-kappaB (NF-κB) activation and may contribute to its transformation capacity. Although Tax-2 has similar protein structure compared to Tax-1, the two proteins display differences both in their protein–protein interaction and activation of signal transduction pathways. Recent studies on Tax-2 have suggested ubiquitylation and SUMOylation independent mechanisms of NF-κB activation. In this present review, structural and functional differences between Tax-1 and Tax-2 will be summarized. Specifically, we will address their subcellular localization, nuclear trafficking and their effect on cellular regulatory proteins. A special attention will be given to Tax-1/Tax-2 post-translational modification such as ubiquitylation, SUMOylation, phosphorylation, acetylation, NF-κB activation, and protein–protein interactions involved in oncogenecity both *in vivo* and *in vitro*.

## INTRODUCTION

Human T cell lymphotropic virus type 1 (HTLV-1) and type 2 (HTLV-2) are closely related human delta retroviruses. Although currently there are four known types of HTLV retroviruses (Mahieux and Gessain, 2005, 2009), HTLV-1 is the most pathogenic of all and the first oncogenic retrovirus discovered in humans. HTLV-1 infects 15–20 million individuals worldwide. It is transmitted horizontally (sexual transmission), vertically (mother to child), and by blood transfusion (Kaplan et al., 1996). HTLV-1 is the causative agent of adult T cell leukemia/lymphoma (ATL; Poiesz et al., 1980a, b; Gallo, 1981; Hinuma, 1982; Hinuma et al., 1982; Yoshida et al., 1982) and tropical spastic paraparesis/HTLV-1-associated myelopathy (TSP/HAM), a distinct neurological disorder with inflammatory symptoms and incomplete paralysis of the limbs (Gessain et al., 1986). HTLV-1 infection is endemic in Japan, Africa, South America, the Caribbean, Melanesia, and certain areas in the Middle East and Eastern Europe (reviewed in Gessain and Mahieux, 2000; Tarhini et al., 2009). The HTLV-1 transactivator protein, Tax-1, has been identified as a significantly potent protein in HTLV-1 pathogenesis. It works as an activator of a variety of transcription factors and has been shown to be sufficient to immortalize T cells *in vitro* and *in vivo* thus playing an important role in cellular transformation (Cereseto et al., 1996; Yao and Wigdahl, 2000; Grassmann et al., 2005; Kashanchi and Brady, 2005; Kfoury et al., 2005; Hasegawa et al., 2006; Mahieux and Gessain, 2007; Matsuoka and Jeang, 2007; Yoshida et al., 2008; Matsuoka and Green, 2009; Yamazaki et al., 2009). HTLV-2, however, was first identified in a T cell line established from a patient with hairy-cell leukemia (Kalyanaraman et al., 1982). In contrast to HTLV-1, HTLV-2 infection has not been linked to the development of lymphoproliferative disorders. However, as in HTLV-1, HTLV-2 infection has been associated with sporadic cases of myelopathy resembling TSP/HAM caused by HTLV-1 (Roucoux and Murphy, 2004). HTLV-2 infection is mainly concentrated in Central and West Africa (Goubau et al., 1990; Gessain et al., 1993), native Amerindian populations in North, Central, and South America (Hjelle et al., 1990; Lairmore et al., 1990; Heneine et al., 1991; Levine et al., 1993), and among intravenous drug users in the United States and Europe (Gazzard et al., 1984; Gallo et al., 1986; Khabbaz et al., 1991; Toro et al., 2005).

## Tax-1 AND Tax-2: THEY LOOK SIMILAR BUT ARE QUITE DIFFERENT

### SEQUENCE AND STRUCTURAL ORGANIZATION

Both Tax-1 and Tax-2 are required for HTLV-1 and HTLV-2 viral replication and they play an important role in proviral transcription (Landry et al., 2007; Yoshida et al., 2008). In addition, Tax-1 is a key player in immortalization and transformation of infected T cells by enhancing the transcriptional expression of genes that control T cell proliferation, affecting genes involved in mitotic checkpoints and further inactivating tumor suppressor pathways (Peloponese et al., 2007; Boxus et al., 2008; Journo et al., 2009; Chlichlia and Khazaie, 2010).

**FIGURE 1:**
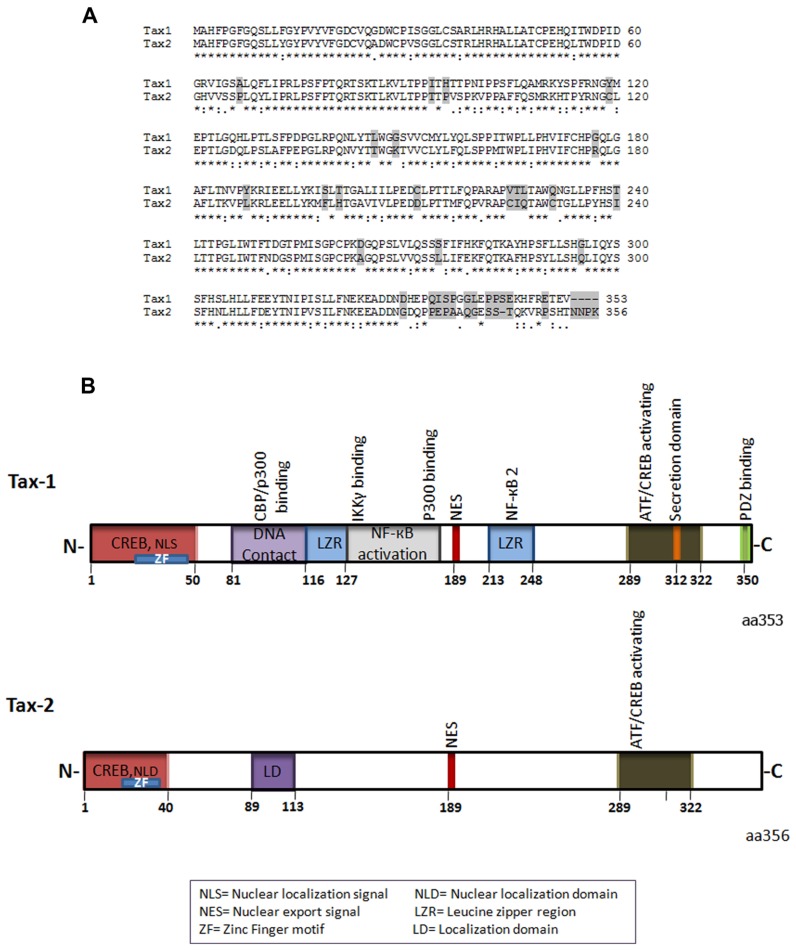
**(A)** Amino acid sequence alignment of Tax-1 and Tax-2 (*) represent identical amino acids, (:) conserved amino acid substitutions, (.) semi-conserved substitutions, differences are shaded. **(B)** Schematic representation of Tax-1 and Tax-2 structural and functional domains.

Tax-1 and Tax-2 share overall sequence homology (**Figure 1A**), but have distinctive differences both at the structural and functional levels (Higuchi and Fujii, 2009; Bertazzoni et al., 2011). Tax-1 is a 353aa (amino acid) residue protein, which is highly conserved in all HTLV-1 serotypes. Of the four serotypes of HTLV-2, Tax-2 subtype A and B are the best characterized (Sheehy et al., 2006) and Tax-2B is the subtype which is represented in **Figure 1**. Tax-2B has 356 amino acid residues, whereas Tax-2A possesses a 25 amino acid truncation at the C-terminus. Tax-1 and Tax-2B share 85% amino acid sequence similarity and have several common domains (**Figure 1A**).

The N-terminal region of both Tax-1 and Tax-2 contain CREB (cyclic AMP responsive element binding)-activating domain and a zinc finger domain (Ross et al., 1997; Feuer and Green, 2005; **Figure 1B**). The CREB domain is required for activation of the viral promoter (Giebler et al., 1997; Boxus et al., 2008). Depending on the cell type, Tax-1 mutants deficient for CREB activation are incompetent for transformation or induction of aneuploidy (Akagi et al., 1997; de la Fuente et al., 2006; Geiger et al., 2008). The zinc finger domain is required for association with a variety of transcription factors including the p62 nucleoporin and mutations in this motif abolishes Tax-1 interaction with p62 and nuclear import (Tsuji et al., 2007). Within the first 60 amino acids of Tax-1, there is a nuclear localization signal NLS (Gitlin et al., 1991; Smith and Greene, 1992) whereas the first 42 amino acid sequence of Tax-2 contain a nuclear localization determinant (Turci et al., 2006) required for its nuclear functionality (**Figure 1B**). Furthermore, Tax-2 has an additional cytoplasmic localization domain about 10 amino acids long, situated at amino acid position 89–113 which has been shown to be responsible for its divergent localization compared to Tax-1 (Meertens et al., 2004).

The central region of Tax-1 includes two leucine zipper-like regions (LZR), which are known to be essential for protein dimerization and DNA interaction (Jin and Jeang, 1997; Basbous et al., 2003; Boxus et al., 2008). The first LZR is located at amino acid position 116–145 and is responsible for non-canonical nuclear factor-kappaB (NF-κB) activation and protein dimerization whereas the second LZR is located at amino acid position 225–232 and is responsible for p100 processing and p52 nuclear translocation involved in NF-κB2 activation (Xiao et al., 2001; Higuchi et al., 2007; Shoji et al., 2009; **Figure 1B**). Importantly, Tax-2 lacks these two LZR regions. Both Tax-1 and Tax-2 have nuclear export signal (NES) located at amino acid position 189–202 (Alefantis et al., 2003; Chevalier et al., 2005; **Figure 1B**). Furthermore, Tax-1 and Tax-2 have at the C-terminal region CREB/activating transcription factor (ATF)-activating domain, essential for transactivation of the CREB/ATF and for NF-κB/Rel signaling pathways (Ross et al., 1997; **Figure 1B**).

Tax-1 interacts *in vitro *with a number of proteins of the CREB/ATF family of transcription factors: CREB, CREM (cyclic AMP responsive element modulator), ATF1, ATF2, ATF3, ATF4 (also named CREB2), and XBP1 (X-box-binding protein 1; Zhao and Giam, 1992; Franklin et al., 1993; Bantignies et al., 1996; Reddy et al., 1997). These proteins share a common cluster of basic residues allowing DNA binding, and a leucine zipper (b-Zip) domain involved in homo and hetero-dimerization. Dimer formation modulates their DNA-binding specificity and transcriptional activity (Hai and Hartman, 2001). Tax-1, but not Tax-2, possesses at its C-terminus a PDZ-binding motif (**Figure 1B**). Indeed, PDZ domain-containing proteins play a key role in recruiting and organizing the appropriate proteins to sites of cellular signaling, as well as polar sites of cell–cell communication (Fanning and Anderson, 1999; Harris and Lim, 2001; Sheng and Sala, 2001). The PDZ domain of Tax-1 has been shown to interact with the human homolog of the *Drosophila melanogaster* disc large tumor suppressor protein hDLG (homolog of *Drosophila* disc large), which regulates cellular proliferation and cell cycle phase transition (Rousset et al., 1998; Higuchi and Fujii, 2009). Tax-1 competes with the binding domain of hDLG and APC (The adenomatous polyposis coli) tumor suppressor protein and rescues cells from cell cycle arrest induced by hDLG (Suzuki et al., 1999; Hirata et al., 2004).

Tax-1 but not Tax-2 contain additionally at the C-terminus a secretory signal which is involved in Tax-1 secretion and transport from endoplasmic reticulum to Golgi and in movement from Golgi to the plasma membrane (Alefantis et al., 2003, 2005). The secretory sequence at the C-terminus requires interaction with secretory carrier membrane proteins (SCAMP-1 and SNAP 23) and the coat protein 2 (COPII; Jain et al., 2007). Recently, the C-terminus of Tax-1 has received much attention due to the presence of domains that are unique for Tax-1 and may partially explain the highest transformation capacity of Tax-1 in comparison to Tax-2. Indeed, the C-terminal 53 amino acids of Tax-1 is responsible for increased transformation efficiency in rodent fibroblasts (Majone et al., 1993).

### CELLULAR LOCALIZATION OF Tax-1 AND Tax-2

Early studies on Tax-1 and Tax-2 subcellular localization have demonstrated that Tax-1 localizes in the nucleus and Tax-2 in the cytoplasm of HTLV-infected cells (Semmes and Jeang, 1996; Meertens et al., 2004). Both Tax-1 and Tax-2 contain a nuclear localization signal at the N-terminus, however, Tax-2 contains an additional cytoplasmic localization domain at position 89–113. By using series of Tax-1/Tax-2 chimeras, Meertens et al. (2004) have shown that this stretch of sequence indeed contributes to the difference in Tax-2 cytoplasmic localization compared to Tax-1.

In various Tax-1/Tax-2 transfected cells lines, Tax-1 has a punctate nuclear distribution and localizes in nuclear structures named nuclear speckles or bodies (Semmes and Jeang, 1996; Bex et al., 1997), whereas Tax-2 was predominantly present in the cytoplasm (Meertens et al., 2004). In these nuclear bodies, Tax-1 colocalizes with proteins of the splicing machinery such as splicing factors Sm and SC-35, transcriptional components including the largest subunit of RNA polymerase II and cyclin-dependent kinase CDK8 and with important components of NF-κB such as the two subunits p50 and RelA, as well as the regulatory subunit NEMO of IkappaB kinase (IKK; Bex et al., 1997). Furthermore, recent findings indicate that Tax-1 colocalizes within nuclear bodies with small ubiquitin modifiers (SUMO-1, 2, and 3; Lamsoul et al., 2005; Nasr et al., 2006) and with the SUMO E2 ligase Ubc-9 (Kfoury et al., 2011).

Although Tax-1 has been shown to be chiefly abundant in the nucleus, many studies reported cytoplasmic expression of Tax-1 in both Tax-1 transfected and HTLV-1-infected cell lines (Burton et al., 2000; Cheng et al., 2001). In the cytoplasm, Tax-1 targets IκBα and IκBβ for phosphorylation, ubiquitylation, and proteasome-mediated degradation, promoting the nuclear translocation of NF-κB/Rel proteins and the transcription induction of many cellular genes (Nicot et al., 1998). Within the cytoplasm, Tax-1 localizes in organelles associated with secretory pathways, structures associated to the centrosome or microtubule organizing center (MTOC), and in the cell to cell contact regions termed virological synapses (Igakura et al., 2003; Alefantis et al., 2005; Kfoury et al., 2008; Nejmeddine et al., 2009). In contrast, Tax-2 has been shown initially to be mostly cytoplasmic with no clear evidence for localization in nuclear bodies (Meertens et al., 2004). However, a recent study reported Tax-2 punctate distribution in nuclear bodies and colocalization with the Rel A subunit of NF-κB (Turci et al., 2006, 2009).

Interestingly, the post-translational modifications of Tax-1 control its sub cellular localization and its ability to activate the NF-κB pathway. More specifically, Tax-1 is subjected to multiple post-translational modifications such as phosphorylation (Bex et al., 1999), ubiquitylation, SUMOylation (Chiari et al., 2004; Lamsoul et al., 2005; Nasr et al., 2006), and acetylation (Lodewick et al., 2009). Ubiquitylated Tax-1 binds and recruits the IKK subunits at a centrosome-associated signalosome leading to the release of active IKK (Nasr et al., 2006; Kfoury et al., 2008). Using live-cell imaging, Kfoury et al. (2011) also showed that Tax-1 shuttles between nuclear bodies and the centrosome, depending on its ubiquitylation and SUMOylation status.

Finally, Tax-1 interacts with histone methyltransferase (HMTase) SMYD3 which affects its nucleo-cytoplasmic shuttling and regulates NF-κB activation (Yamamoto et al., 2011). Interaction of Tax-1 with the four and a half LIM domain protein 3 (FHL3) also affects Tax-1 sub cellular localization and transactivation capacity (Mccabe et al., 2013).

## MODULATION OF CELLULAR PATHWAYS BY Tax-1 AND Tax-2

Tax-1 interacts with various components of the cell signaling system which control cell transformation, proliferation, intracellular protein distribution, cell migration, and virological synapses (Azran et al., 2004; Jeang et al., 2004; Grassmann et al., 2005; Boxus et al., 2008). More than 100 proteins have been reported to interact with Tax-1 (Boxus et al., 2008). Tax-2, however, interacts with a limited number of partners and most of them belong to the NF-κB family of proteins. It is important to note that Tax-1 and to a lesser extent Tax-2 interactome is undergoing a dramatic expansion with additional interaction partners being discovered continuously.

### PI3K AND AKT PATHWAY

Phospho-inositol triphosphate kinase (PI3K) and its downstream kinase AKT/PKB (protein kinase B) are activated in T cells by many cytokines including interleukin 2 (IL-2), and provide cell survival and growth signals (Cantley, 2002). PI3K activation results in phosphorylation of AKT at Ser^473^ which in turn activates a broad range of regulatory proteins and transcription factors such as AP1 (Zhang et al., 2007). In both HTLV-1 transformed and ATL cells, the transcription factor AP1 and hence the PI3K/AKT pathway are constitutively active (Fukuda et al., 2005; Peloponese and Jeang, 2006). The PI3K inhibitor (LY294002) or the AKT inhibitor II were shown to induce cell cycle arrest at G1 phase in HTLV-1 transformed cells through p27/Kip1 accumulation, and thus subsequently induce caspase-9-dependent apoptosis (Jeong et al., 2008). Other studies have shown an important role for PI3K/AKT pathway in regulating telomerase activity, and inhibition of PI3K decreased telomerase activity by more than 50% in HTLV-1-infected cells (Bellon and Nicot, 2008). Tax-1 has been also shown to be involved in Forkhead Box O (FoxO) down regulation, an AKT downstream effector and a tumor suppressor, through the ubiquitin–proteasome pathway (Oteiza and Mechti, 2011). Conversely, a recent study demonstrated that Tax-2 efficiently immortalized human primary CD4^+^ memory T cells by constitutively activating various signaling pathways including the PI3K/AKT pathway and further found that Tax-2 induced autophagy by interacting with the autophagy complex that contains Beclin1 and PI3K class III to form autophagosomes (Ren et al., 2012).

### MAPK SIGNALING PATHWAY

Mitogen-activated protein kinases (MAPKs) are serine/threonine-specific protein kinases that respond to external mitogen stimuli such as growth factors, cytokines or physical stress. MAPK signaling involves a sequential phosphorylation cascade of MAP kinase kinase kinase (MAP3K). There are at least five distinct MAPK subgroups: the extracellular signal-regulated kinases protein homologs 1 and 2 (ERK1/2), the big MAPK-1 (BMK-1) also referred to as ERK5, the stress-activated protein kinases-1 (SAPK-1) better known as the c-Jun N-terminal kinase homologs 1, 2, and 3 (JNK1/2/3), the (SAPK-2) homologs (p38α/β/d) and finally ERK6 also known as p38γ (Pimienta and Pascual, 2007). Tax-1 binds the MAP3K MEKK1 to stimulate IKK-β kinase activity and NF-κB activation (Yin et al., 1998). TGF-β -activating kinase 1 (TAK1) is the other MAP3K which interacts with Tax-1 and phosphorylates IKK-β and MKK6 (MAP2K6) serine/threonine kinase, thereby activating NF-κB and JNK (Adhikari et al., 2007). Tax-2 interaction with the MAPK signaling pathway leading to its constitutive activation have also been recently reported (Ren et al., 2012).

### TGFβ SIGNALING PATHWAY

Transforming growth factor β (TGFβ) inhibits T cell growth in mid-G1 but can also promote tumorigenesis (Pennison and Pasche, 2007). TGFβ binds to a heterodimeric complex composed of type I (TβRI) and type II (TβRII) serine/threonine kinase receptors and activates downstream targets such as Smad proteins. These include receptor-activated R-Smad (Smad1–2–3–5–8) and the common mediator Co-Smad (Smad4). Smad4 containing complexes then translocate to the nucleus and activate transcription of genes under the control of a Smad-binding element (Waterston and Davies, 1993).

Adult T cell leukemia/lymphoma cells produce high levels of TGFβ in the sera of HTLV-1-infected patients due to constitutive activation of AP-1 in the PI3K/AKT pathway (Kim et al., 1990). Tax-1 binds the N-terminus of Smad2, Smad3, and Smad4 proteins, which inhibits their association with Smad-binding elements and competes with Smads for recruitment of CBP/P300. This inhibition will also result in promoting resistance of HTLV-1-infected cells to TGFβ (Mori et al., 2001; Arnulf et al., 2002; Lee et al., 2002). So far, interaction of Tax-2 with Smads has not been reported.

### G PROTEINS AND CYTOSKELETAL ORGANIZATION

The guanine nucleotide-binding proteins GTPases (G proteins) are molecular switches that cycle between active (GTP-bound) and inactive (GDP-bound) states. Tax-1 forms complexes with several members of the small GTPase Rho family G proteins such as RhoA, Rac, Gap1m, and Cdc42 (Wu et al., 2004). Rho GTPases are activated in response to external stimuli such as growth factors, stress, or cytokines. Following activation, they regulate a variety of cellular and biochemical functions such as cytoskeleton organization, regulation of gene expression, and enzymatic activities (Jaffe and Hall, 2005).

Tax-1 binds to proteins involved in cytoskeleton structure and dynamics such as α-internexin, cytokeratin, actin, gelsolin, annexin, and γ-tubulin (Trihn et al., 1997; Reddy et al., 1998; Wu et al., 2004; Kfoury et al., 2008) and through these interactions it might connect Rho GTPases to their targets and affects cytoskeletal organization. Tax-1 binds the Gβ subunit of the G protein-coupled receptor (GPCR) affecting the SDF-1-dependent activation of CXCR4 GPCR chemokine receptor resulting in MAPK pathway over-activation and increased cell chemotaxis (Ohshima, 2007). Additionally, Tax-1 expression at the microtubule assembly center and the Golgi in the cell to cell contact region has been shown to contribute to the intracellular signal which synergizes with ICAM-1 (intracellular adhesion molecule) to induce T cell microtubule polarization at the virological synapse (Nejmeddine et al., 2005, 2009). Tax-2, however, has not yet been reported to associate with proteins involved in cytoskeletal rearrangement. It is of importance to mention again that Tax-2 lacks a PDZ domain (**Figure 1**). This PDZ domain might contribute to Tax-1 binding to proteins involved in microtubule and cytoskeleton organization, which in turn may play an important role in pathogenicity and transformation capacity (Endo et al., 2002; Ishioka et al., 2006).

### ACTIVATION OF CREB SIGNALING

As mentioned previously, both Tax-1 and Tax-2, respectively, act as transcriptional activators of the HTLV long terminal repeat (LTR). Tax-1 and Tax-2 modulate CREB and ATF function (Jeang et al., 1988; Adya and Giam, 1995; Bodor et al., 1995; Brauweiler et al., 1995; Yin et al., 1995b; Bantignies et al., 1996; Tie et al., 1996; Yin and Gaynor, 1996; Bex et al., 1998). Tax-1/Tax-2 activation of the CREB/ATF pathway is critical for efficient viral gene expression and replication (Zhao and Giam, 1992; Wagner and Green, 1993; Adya et al., 1994; Anderson and Dynan, 1994; Yin et al., 1995a; Bantignies et al., 1996). A number of mutants in both Tax-1 and Tax-2 have been described that selectively abrogate the ability of Tax to activate transcription through the CREB/ATF signaling pathway (Smith and Greene, 1990; Semmes and Jeang, 1992; Ross et al., 1997). Tax-1 activates a variety of cellular genes through its interactions with CREB/ATF proteins, for example those encoding IL-17 or c-fos (Alexandre and Verrier, 1991; Dodon et al., 2004). On the other hand, Tax-1 also represses expression of genes like cyclin A, p53, and c-myb by targeting CREB/ATF factors (Nicot et al., 2000; Kibler and Jeang, 2001). Furthermore, Tax-1 has been shown to repress Smad-dependent TGFβ signaling through interaction with CBP/p300 (Mori et al., 2001). Tax-1 has also been shown to abrogate p53-induced cell cycle arrest and apoptosis through its CREB/ATF functional domain (Mulloy et al., 1998). Some bioinformatic analysis of wild type and CREB-deficient Tax-1 protein revealed several cellular genes controlled by CRE elements activated by Tax-1 (de la Fuente et al., 2006) such as Sgt1 (suppressor of G2 allele of SKP1) and p97 (Vcp; valosin containing protein) which have functions in spindle formation and disassembly, respectively.

Both Tax-1 and Tax-2 interact with a series of CREB/ATF factors and modulate expression of viral and cellular genes through CRE elements. However, the specific binding of each CREB/ATF member still needs to be studied, although some in vitro analysis suggest Tax-1 interaction with a number of proteins of the CREB/ATF family of transcription factors: CREB, CREM, ATF1, ATF2, ATF3, ATF4 (also named CREB2), and XBP1 (Zhao and Giam, 1992; Franklin et al., 1993; Bantignies et al., 1996; Reddy et al., 1997).

### REPRESSION OF P53 SIGNALING

P53 is a DNA-binding transcription factor, which plays an important role as a tumor suppressor and is primarily involved in cell cycle regulation, apoptosis, and DNA repair (Vousden and Lu, 2002; Zenz et al., 2008). The P53 gene is very often mutated in human tumors and hematologic malignancies (Xu-Monette et al., 2012). Several *in vitro* studies in different cell types have shown that Tax-1 represses p53 activity through different mechanisms including NF-κB activation and/or the CREB pathway (Ariumi et al., 2000; Pise-Masison et al., 2000; Jeong et al., 2004, 2005). Recently, Wip-1 phosphatase protein was shown to interact with Tax-1 and inhibits p53 (Zane et al., 2012). In this study authors have used Tax transgenic mice and found significant differences in Tax-1-driven inactivation of p53 versus p53 inactivation due to genetic mutations. Several studies explored Tax-2 contribution to p53 inactivation. In HTLV-2 subtype A- and B-infected cells, both Tax-2B and to a lesser extent Tax-2A were shown to inhibit p53 in T cells (Mahieux et al., 2000b).

In ATL-derived cell lines, P53 has been shown to be very often inactive and sometimes mutated despite its high expression levels and this activation has been shown to be dependent on Tax-1-induced NF-κB activation through phosphorylation of p53 Ser-15 and Ser-392 (Pise-Masison et al., 2000). Studies by Ariumi et al. (2000) have shown that the phosphorylation of p53 on Ser-15 is not a major cause of the Tax-mediated inactivation of p53. However, Tax with a mutation in the coactivator CBP-binding site (K88A), which activates NF-κB but not the CREB pathway, could not repress the p53 transactivation function. A study dedicated to Tax-2 inhibition of p53 was performed by (Mahieux et al., 2000a) where abundant levels of p53 protein were detected in both HTLV-2A and -2B virus-infected cell lines and p53 was shown to be inactive. Furthermore, they showed that although Tax-2A and Tax-2B inactivate p53, the Tax-2A protein appeared to inhibit p53 function less efficiently than either Tax-1 or Tax-2B. Jurkat cells that constitutively express Tax-1 and Tax-2 showed reduced cellular replication, and Tax-1 inhibition of cellular replication was higher in comparison to Tax-2 (Sieburg et al., 2004).

### ACTIVATION OF THE NF-κB PATHWAY

#### Generalities on NF-κB

Nuclear factor-kappaB is a family of transcription factors that play a crucial role in proliferation, apoptosis, oncogenesis, and immune response. To date, five members of NF-*κ*B have been described: p65 (RelA), c-Rel, RelB, p50/p105, and p52/p100. The precursor proteins p105 and p100 are processed proteolytically to the mature p50 and p52 forms, respectively (Ghosh and Hayden, 2008). All five members share a common Rel homology domain, which is a conserved domain of 300 amino acids that contains a DNA-binding domain, a dimerization domain, a region of interaction with inhibitory proteins IκB, and a NLS (Baeuerle and Henkel, 1994; Baldwin, 1996). These proteins are capable of homo- or heterodimerization using all possible combinations, except for RelB which dimerizes only with p50 or p52 (Ryseck et al., 1992).

In resting cells, NF-κB dimers are trapped in the cytoplasm by inhibitory proteins called IκBs such as p105, p100, IκBα, IκBβ, and IκBγ which mask the nuclear localization signal of NF-κB factors through physical interaction (Siebenlist et al., 1994; Perkins, 2007). NF-κB activation involves phosphorylation of IκB inhibitors by the IKK, which triggers their ubiquitylation and subsequent proteasomal degradation, resulting in nuclear translocation of NF-κB dimers (Karin and Ben-Neriah, 2000; Perkins, 2007).

Nuclear factor-kappaB is activated by a wide variety of signals through two distinct pathways: the canonical and the non-canonical pathways. The canonical pathway is activated by pathogens, cytokines, and antigen receptors and involves the degradation of one of the three canonical IκB molecules: IκB-α, IκB-β, and IκB-ε and the nuclear translocation of the heterodimers that essentially contain RelA (Silverman and Maniatis, 2001). In response to activating signal, the IκB proteins are phosphorylated by the IKK complex, which is a high molecular weight complex composed of one regulatory subunit IKK-γ (NEMO) in addition to two catalytic subunits IKK-α and IKK-β (Israel, 2010). Upon activation, the IKK complex is able to induce the phosphorylation of the IκB proteins leading to their ubiquitylation and degradation by the proteosome. The non-canonical NF-κB pathway on the other hand primarily involves IKK-α activation upon phosphorylation by NF-κB-inducing kinase (NIK). IKK-α then phosphorylates the C-terminal region of p100 leading to subsequent processing of the p100/RelB complex into p52/RelB and its translocation into the nucleus (Dejardin, 2006). It is important to note that p52/RelB and p50/RelA dimers target distinct NF-κB enhancers thereby activating different subset of genes.

#### Tax-1 activation of the NF-κB pathway

**FIGURE 2:**
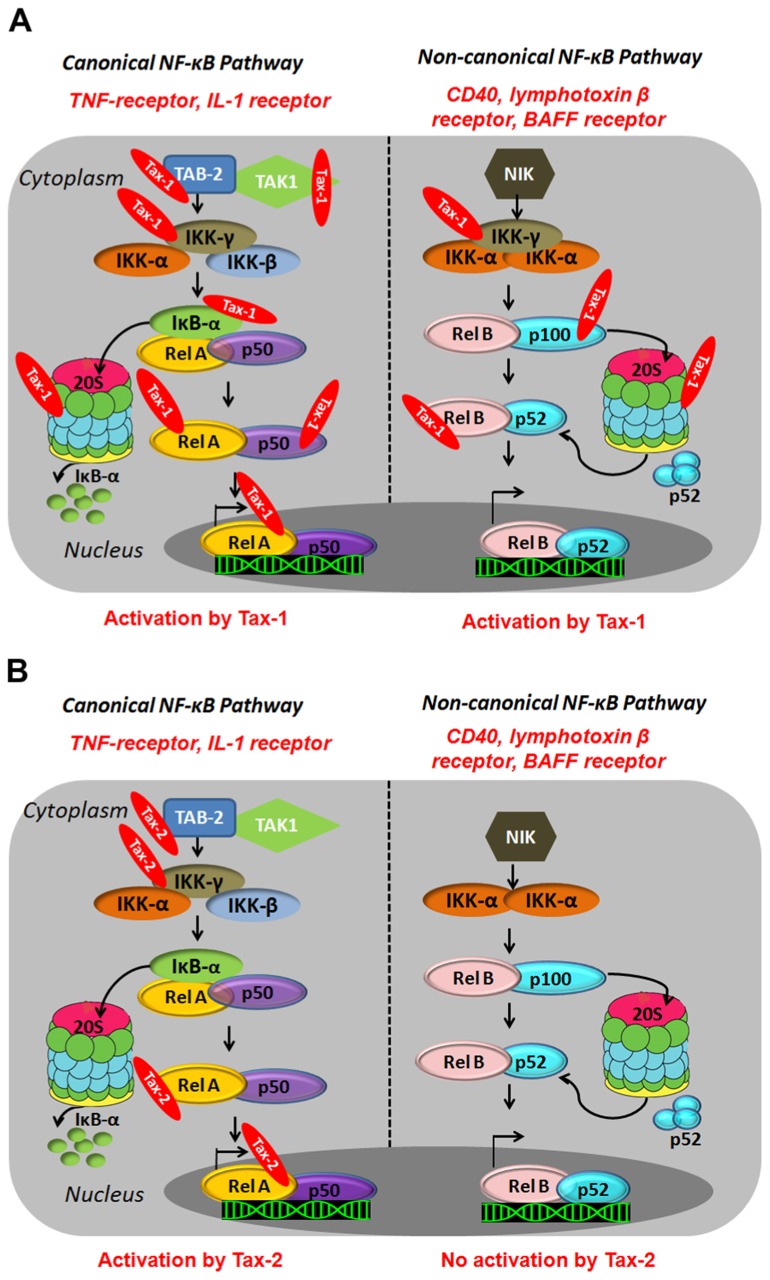
**Illustration of canonical and non-canonical NF-κB pathway activation by HTLV-1 Tax-1 **(A)** and Tax-2 **(B)**.** Canonical NF-κB pathway involves a cascade of phosphorylation events by kinases such as TAK1 and the IKK complex (α,β,γ) which results in the proteasomal degradation of the cytoplasmic inhibitor (IκB) and the translocation of the NF-κB dimers to the nucleus and transcriptional activation. The non-canonical pathway involves the NF-κB-inducing kinase (NIK) and IKK-α subunit (α) and results in the proteasomal degradation of p100 and the nuclear translocation of NF-κB dimers p52/RelB and activation of gene expression.

Tax-1 activates both the canonical and the non-canonical pathways resulting in constitutive activation of NF-κB in HTLV-1-infected cells (Xiao et al., 2001; Higuchi et al., 2007). In the canonical pathway, Tax-1 associates with the IKK-γ/NEMO subunit (Harhaj and Sun, 1999; Jin et al., 1999; Kfoury et al., 2005) and activates upstream kinases such as MAPK/ERK kinase kinase 1 (MEKK1), and TAK1 through TAK1-binding protein 2 (TAB2; Yin et al., 1998; Wu and Sun, 2007; **Figure 2A**). Tax-1 therefore, connects activated kinases to the IKK complex and forces the phosphorylation of IKK-α and IKK-β leading to IKK activation, which results in phosphorylation, ubiquitylation, and proteasome-mediated degradation of IκBα and IκBβ (Harhaj and Sun, 1999; Jin et al., 1999). In addition, Tax-1 binds directly to the IKK-α and IKK-β subunits and activates their kinase activity independently of the upstream kinases (Chu et al., 1998; **Figure 2A**). In fact, silencing of MEKK1 and TAK1 does not impair Tax-1-induced NF-κB activation (Gohda et al., 2007). Within the canonical pathway, Tax-1 can as well bind directly to IκBs and mediate their degradation independently of IKK phosphorylation (Hirai et al., 1994; Suzuki et al., 1995). At the proteosomal level, Tax-1 interacts with the two subunits of the 20S proteasome (HsN3 and HC9), favors anchorage of p105 and accelerates its proteolysis (Rousset et al., 1996; **Figure 2A**). Tax-1 therefore, leads to IκB degradation at multiple levels, thereby allowing nuclear translocation of NF-κB independently of external stimuli. In the non-canonical pathway, Tax-1 interacts with IKK-γ (NEMO) and p100, induces p100 processing and nuclear translocation of the p52/RelB dimer (**Figure 2A**). It therefore appears that IKK-γ is an important Tax-1-binding partner for activation of both pathways (Xiao et al., 2001; Higuchi et al., 2007).

#### Tax-2 activation of the NF-κB pathway

Many studies have shown the ability of Tax-2 to activate the canonical NF-κB pathway to a level comparable to Tax-1 (Higuchi et al., 2007). The major difference between Tax-1 and Tax-2 lies in the inability of Tax-2 to process p100 (Higuchi et al., 2007; **Figure 2B**). The LZR at amino acid 225–232 of Tax-1, which is missing in Tax-2, is responsible for p100 processing and p52 nuclear translocation (Shoji et al., 2009). To date, there is no evidence of the ability of Tax-2 to activate the non-canonical NF-κB pathway. In fact, the transforming activity of Tax-1 in CTLL-2 (cytotoxic T-lymphocyte cell lines) cells constitutively expressing the IL-2 receptor is much higher than Tax-2 and this activity has been shown to be partly mediated through the non-canonical NF-κB pathway (Tsubata et al., 2005; Kondo et al., 2006; Higuchi et al., 2007; Shoji et al., 2009). Within the same line, a constitutively active NIK, restores the transforming activity of Tax-2 to a level equivalent to Tax-1 (Higuchi et al., 2007). This inability of Tax-2 to activate the non-canonical NF-κB pathway might partially explain its inability to transform T cells and induce ATL development.

## Tax-1 AND Tax-2 POST-TRANSLATIONAL MODIFICATIONS

Post-translational modifications of Tax-1 and Tax-2 proteins have been shown to play a critical role in their cellular localization, transactivation, and protein–protein interactions. Furthermore, Tax-1 and Tax-2 pleotropic effects and their structural organization make these proteins a target of many other potential post-translational events which still need to be discovered.

### PHOSPHORYLATION

**FIGURE 3:**
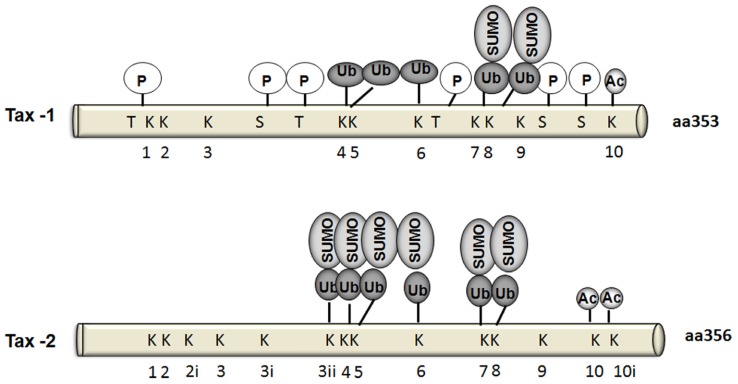
**Schematic comparison of Tax-1 and Tax-2 post-translational modifications.** Sites of phosphorylation (P), and target lysines for ubiquitylation, SUMOylation, or acetylation are indicated.

To date, six Tax-1 residues were identified as phosphorylation targets: Thr-48, Thr-184, Thr-215, Ser-300, Ser-301, and Ser-336 (Bex et al., 1999; Durkin et al., 2006; **Figure 3**). Adjacent serine residues at positions 300 and 301 in the carboxy-terminus of Tax represent the major sites for phosphorylation. Indeed, phosphorylation of at least one of these serine residues is required for Tax localization in nuclear bodies and for Tax-mediated activation of gene expression via both the ATF/CREB and NF-κB pathways (Bex et al., 1999). Furthermore, Ser-300 and Ser-301 are required for further post-translational modifications such as ubiquitylation, SUMOylation, and acetylation (Lodewick et al., 2009). On the other hand, the serine/threonine kinase CK2 phosphorylates Tax-1 at three residues: Ser-336, Ser-344, and Thr-351 within its C-terminus, which indirectly affects NF-κB activation (Higuchi et al., 2007; Bidoia et al., 2010). Some indirect evidence of the involvement of Ser-160 phosphorylation in stabilizing Tax-1 has been recently reported (Jeong et al., 2009). Although Tax-1 and Tax-2 share 85% homology in their amino acid sequences, and all the phosphorylated residues are conserved except for Ser-336, the phosphorylation status of Tax-2 is still not well determined. *In vitro* studies showed that CK2 does not phosphorylate Tax-2 as for Tax-1 (Bidoia et al., 2010). A detailed mutational analysis of Tax-2 residues may help in identifying Tax-2 phosphorylated residues and their impact on Tax-2 function.

### ACETYLATION

Tax-1 has been shown to be acetylated at Lys-346 (Lodewick et al., 2009). Acetylated forms of Tax-1 were detected in both Tax-1 transfected 293 T cells and T lymphocytes (Lodewick et al., 2009). In the same study it has been suggested that phosphorylation of Ser-300/Ser-301 is essential for its nuclear translocation and hence is a prerequisite for Tax-1 acetylation through interaction with p300 (**Figure 3**). Tax-1 acetylation in turn participates in NF-κB activation (Lodewick et al., 2009). Although there is not much studies yet on Tax-2 acetylation, Lodewick et al. (2009) reported that Tax-2 may also be acetylated.

### UBIQUITYLATION AND SUMOylation

Ubiquitylation and SUMOylation have been shown to play an important role in the cellular localization, function, and protein–protein interactions of both Tax-1 and Tax-2 (Chiari et al., 2004; Peloponese et al., 2004; Harhaj et al., 2007; Turci et al., 2009; Avesani et al., 2010). Tax-1 has ten lysines (**Figure 3**). Five of these residues located within Tax-1 C-terminal region were found to be the major targets ubiquitylation [Lys-189 (K4), Lys-197 (K5), Lys-263 (K6), Lys-280 (K7), and Lys-284 (K8)], whereas SUMOylation takes place on Lys-280 (K7) and Lys284 (K8) (Lamsoul et al., 2005; Nasr et al., 2006).

Tax-1 is indeed differentially ubiquitylated by either K-48 ubiquitin chains leading to Tax degradation by the proteasome or by K-63 ubiquitin chains that mediates IKK recruitment to the centrosome and IKK activation (Kfoury et al., 2008). On the other hand, Tax-1 SUMOylation is required for nuclear body formation and recruitment of RelA and IKK-γ to Tax-1-related nuclear bodies, where Tax-driven transcription is promoted (Lamsoul et al., 2005; Nasr et al., 2006; Harhaj et al., 2007; Kfoury et al., 2011). A RING (Really Interesting New Gene) finger domain containing protein RNF4 has recently been shown to bind putative Tax ubiquitin/SUMO modification sites K280/K284 and increase Tax cytoplasmic enrichment and NF-κB activation (Fryrear et al., 2012). A recent report added new insights to our understanding of Tax-1 and Tax-2 ubiquitylation- and SUMOylation-dependent NF-κB activation. Bonnet et al. (2012) used Tax-1 mutants (Tax-P79AQ81A) defective for nuclear body formation. Ubiquitylation levels of the mutant and the wild type protein were similar, however, the endogenous SUMOylation levels were lower in the mutant. Despite low SUMOylation levels in the mutants, NF-κB activation was not affected enforcing the possibility that low levels of SUMOylation may suffice for Tax-1-induced NF-κB activation.

The involvement of Tax-2 SUMOylation and ubiquitylation in NF-κB activation remains controversial. Journo et al. (2013) showed that in contrast to Tax-1, Tax-2 SUMOylation and ubiquitylation are not essential to activate NF-κB. In their study, Tax-2 conjugation to endogenous SUMO and ubiquitin was barely detectable, however, Tax-2 was still acetylated. This low level of conjugation to endogenous ubiquitin and SUMO did not prevent Tax-2 activation of an NF-κB-dependent promoter or its interaction with IKK-γ/NEMO. Furthermore, a lysine-less Tax-2 mutant, which is defective for ubiquitylation and SUMOylation but not acetylation, is still able to transactivate an NF-κB-dependent promoter and bind and activate the IKK complex to induce RelA/p65 nuclear translocation. On the other hand, using transfection methods, Turci et al. (2012) have reported that Tax-1 and Tax-2 share a common mechanism of NF-κB activation and that both depend on their ubiquitylation and SUMOylation status. Thus, they show that patterns and levels of ubiquitylation between Tax-1 and Tax-2 are conserved, except for a reduced representation of the Tax-2 mono-ubiquitylated form compared to Tax-1.

## INHIBITION OF APOPTOSIS AND INDUCTION OF DNA DAMAGE BY Tax-1 AND Tax-2

Induction of programmed cell death by Tax-1 has been shown in many studies using both *in vitro* Tax-1 inducible cell lines (Ray and Gottlieb, 1993) and *in vivo* transgenic mice. Indeed, Tax-1 transgenic mice are characterized by enhanced apoptosis which is associated with elevated levels of oncoproteins such as Myc, Fos, Jun, and p53 expression (Hall et al., 1998). It is important to mention that ATL malignant transformation involves complex and multi-step mechanisms such as accumulation of DNA damage and aneuploidy. Furthermore, Tax-1 expression sensitizes cells to apoptotic cell death induced by DNA damaging agents (Kao et al., 2000) and by tumor necrosis factor alpha (TNF-α; Saggioro et al., 2001). Upon UV irradiation, Tax-1 localization was increased at the cytoplasm and decreased in the nucleus and Tax-1 NES have been shown to be required for its stress-induced nucleocytoplasmic translocation (Gatza and Marriott, 2006). Caspase activity has been shown to be crucial for Tax-1-induced cell death and apoptosis whereas B cell lymphoma 2 (Bcl-2) expression has been shown to be associated with cell death prevention (Yamada et al., 1994; Chen et al., 1997; Chlichlia et al., 1997, 2002; Rivera-Walsh et al., 2001; Kasai and Jeang, 2004). Interestingly, Tax has been shown by many studies to both induce apoptosis and represses it. Many groups have shown the importance of Tax-1-mediated NF-κB activation in induction of apoptosis (Wheeler et al., 1993; Chen et al., 1997; Chlichlia et al., 1997; Los et al., 1998; Rivera-Walsh et al., 2001). Tax mutants defective in NF-κB activation have reduced apoptosis-inducing activities, and inhibition of Tax-mediated NF-κB transactivation partially inhibited apoptotic cell death (Los et al., 1998; Harrod et al., 2000; Rivera-Walsh et al., 2001). Tax also represses the transcription of the proapoptotic *bax* gene (Brauweiler et al., 1997). In addition, Tax inhibits the caspase cascade in an NF-κB-dependent manner through the induction of the caspase inhibitors X-IAP, cIAP-1, and c-IAP-2 (Kelly et al., 1993).

Previous experiments performed on T cell lines derived from HTLV-2-infected individuals and Tax-2 expressing various cell lines have shown that Tax-2 is capable of inhibiting Fas-mediated apoptosis through the expression of bcl-x(L) messenger and protein (Zehender et al., 2001).

## CONCLUDING REMARKS

To date, vast amount of knowledge has been produced regarding the HTLV-1 Tax-1 oncoprotein. Many studies have provided some insights on Tax-1 transcriptional regulation, subcellular localization and post-translational modifications. However, less is known about HTLV-2 Tax-2 although many aspects of its activity and regulation is now being studied. That HTLV-2 is defective in promoting certain steps of leukemogenesis, may indeed serve as a useful comparative tool for understanding the pathogenicity of HTLV-1.

## Conflict of Interest Statement

The authors declare that the research was conducted in the absence of any commercial or financial relationships that could be construed as a potential conflict of interest.
